# Building the Machine: The Importance of Governance in Obesity Policy

**DOI:** 10.3389/fpubh.2018.00221

**Published:** 2018-08-03

**Authors:** Andrew J. Pengilley, Paul M. Kelly

**Affiliations:** ^1^Population Health Division, ACT Health, ACT Government, Canberra, ACT, Australia; ^2^Australian National University Medical School, Canberra, ACT, Australia; ^3^The Australian Prevention Partnership Centre, Sax Institute, Sydney, NSW, Australia

**Keywords:** obesity, government, overweight, governance, policy

## Abstract

The Australian Capital Territory (ACT) is a small Australian jurisdiction with a single tier of government and a population of approximately 400,000 people. Despite enjoying comparatively high levels of income, education, physical amenity, and access to nutritious food, overweight and obesity is the most prevalent risk factor for chronic disease in the ACT. From 2011, the ACT Government Health Directorate (ACT Health) led the development of a whole of Government plan (the Action Plan) to address obesity. A political imperative to take such action and recent administrative reform assisted the development of a plan with specific actions to be undertaken by different government agencies. Obesity is a “wicked problem” with a diversity of opinion about its causes and potential solutions. These opinions remained influential even when an official course of action had been decided upon. Strong decision making and accountability processes were therefore necessary to support the development of the Action Plan. A lack of understanding beyond the health sector in relation to the evidence for effective, population level interventions to address obesity and a tendency to try and address population health risks by scaling up client-centered models of Government services also proved problematic. This experience highlights the critical importance of designing obesity policy within a robust governance framework in order to ensure progress is made in a highly contested environment. Whilst the observations included here are strongly influenced by local contextual factors, there are important lessons which can be applied elsewhere.

## Introduction

Overweight and obesity is the most prevalent chronic disease risk factor in the Australian Capital Territory (ACT), affecting two-thirds of adults and one quarter of children (1). These rates have increased dramatically over the past 25 years, despite the ACT having extensive green space, a relatively well-educated and affluent population and access to high quality food (1). While there is a socio-demographic gradient in the prevalence of overweight and obesity in the ACT, the fact that the majority of the population is affected suggests that the obesity epidemic is not caused by socio-economic determinants alone.

Reducing rates of overweight and obesity is a government priority due to the associated increased risk of chronic illness and the significant health services burden (2). It is widely recognized that effective action requires collaboration beyond the health sector in areas such as transport, planning and education (3). The theoretical frame for cross-sectoral approaches to wicked problems in health has been documented by de Leeuw (4). She argues that, since the conceptualization of “healthy public policy” more than 30 years ago, most analyses have remained abstract, focused on arguing the case for the approach rather than showing how it can be done and grounding these examinations in political theory. This paper presents a practical, real world example of where such a cross-sectoral approach to the “wicked problem” of obesity was achieved at the local level.

## Method

A reflective practice method (5) was employed to document and analyze the key components of the lived experience of the authors, who were participant-observers tasked with leading the development of the *Toward Zero Growth: Healthy Weight Action Plan (the Action Plan)* (6). From the initiation of this work in February 2011 to the public launch of the Action Plan in October 2013, the authors and other members of the steering group within ACT Health met at least weekly to discuss progress and to plan the next steps. At each meeting, notes were made and key learnings as well as progress were documented. In preparation for the paper, these notes were reviewed and major themes were extracted, compared, refined and then triangulated with the authors' individual reflective notes, as well as those of other members of the steering group. External researchers involved with qualitative studies of the related ACT Healthy Weight Initiative also reviewed the paper and provided valuable insights.

## The Australian capital territory: local context

The ACT is a small jurisdiction in south-eastern Australia in which Canberra, a low density urban area (160 people/km^2^) with a population of approximately 412,000 people is located (7). Uniquely in Australia, the ACT has one tier of government, with both local municipal and provincial level responsibilities.

## Whole of government action: the authorizing environment

Under the ACT *Public Health Act 1997*, the Chief Health Officer is required to report on trends and indicators in health status for the ACT population (8). The *2010 Chief Health Officer's Report* indicated a high prevalence of overweight and obesity across the life-course and shortly after the publication of this report (9), the then Health Minister requested that the Chief Health Officer develop a “whole of government response” to this issue.

The initial approach to this was to write to the administrative heads of all ACT Government agencies and request information about what actions their respective agencies were taking to address obesity. The responses reinforced the impression that other agencies considered obesity to be a “health agency problem”.

Whole of government action to address obesity may well have stopped there, but for two important developments in the authorizing environment to support coordinated action in early 2011. Firstly, the Health Minister became the head of government (Chief Minister). Secondly, a comprehensive review of the ACT Public Service (ACTPS) was undertaken, examining its capacity to support strategic advice, and to coordinate cross-government service delivery (10). The review recommended a range of reforms to centralize strategic direction including the establishment of a strategic board to be comprised of all agency heads and chaired by the head of the Chief Minister's directorate (10).

Having a new Chief Minister (who was also the Health Minister) who was passionate about addressing obesity, together with this new bureaucratic structure for whole-of-government collaboration presented a unique window of opportunity. Centralized authority provided an opportunity to resolve competing government agendas, and reduced the complexity of managing multi-agency action.

However, this level of political support imposed an almost immediate expectation to provide tangible outcomes to justify the Government's focus on obesity. This significantly constrained the length and scope of consultation which could be undertaken before proposing specific actions. Additionally, it did not allow time for incremental change in the culture and understanding of colleagues from non-health backgrounds. The Chief Minister was clear, however, that this work must not merely result in a “forgettable summit,” nor produce a policy document to sit on a shelf. Rather, the aim was to design a suite of new initiatives, coordinated across government, to meaningfully contribute to halting the rise in the rate of overweight and obesity.

## Addressing obesity: which model to choose?

The development of the Action Plan was informed by two existing models already operating in other Australian states; whole of government strategic plans and Health in All Policies.

### Whole of government strategic plan

In 2011, two Australian states had well-developed whole of government strategic plans auspiced by their respective Premier's Department, namely *Toward Q2: Tomorrow's Queensland* and *South Australia's Strategic Plan* (11, 12). These plans specified mutually supportive key performance indicators and targets for health and other agencies within their government structures.

The strength of this approach was to embed a clear element of co-production for co-benefit across portfolios. The non-health agencies had relevant performance targets set in their own areas of responsibility which in turn provided strong anchor points for collaboration on health issues. For example, an environmental agency tasked with reducing carbon emissions could collaborate with a transport agency tasked with increasing use of public transport, which in turn provided an opportunity to leverage active transport to address obesity. Discussions with departmental officers in these states indicated that over several years, cross-agency advocacy and collaboration became entrenched in the process of bidding for human and financial resources, thereby further enhancing cross-agency cooperation.

This approach was proposed for the Action Plan but was not adopted by the Strategic Board. This was primarily due to the absence, at that time, of an overarching plan which gave sufficient policy clarity in areas other than obesity prevention.

### Health in all policies

The World Health Organization endorsed Health in All Policies (HiAP) approach has been extensively evaluated in a range of settings (4). The South Australian Government established a HiAP unit in 2007 (13) and ran in tandem with the implementation of *South Australia's Strategic Plan* by applying a “health lens” to policies across government. In theory, through the process of looking at policy in terms of what it could contribute to health outcomes, or what barriers it may present to achieving desired health outcomes, the policy environment could be reoriented to be health promoting. This is consistent with the Ottawa Charter which places “building healthy public policy” as a core aim of health promotion (14). HIAP advocates have emphasized the need for strong governance and centralized authority to effectively drive policy reform—something that *South Australia's Strategic Plan* provided.

The establishment of a HIAP unit in the ACT context was considered, but the likely utility of such a unit was deemed to be limited. Indeed, the aforementioned review of the ACTPS noted.
“*There was a clear and consistent view in consultation that the ACT Government has too many plans, leading to a propensity for the ACTPS to ‘tie itself in knots' with snowballing layers of plans, strategies, action plans, implementation plans, statements of intent, frameworks and performance agreements” (10)*.

Our experience suggested that official policy statements do not necessarily reflect the power relationships or depths of division which determine what gets done in government. The problem is not necessarily unhealthy policy *per se*, but the effectiveness of the policy process to ensure that competing priorities and resource limitations are addressed. It was therefore considered important to embed the policy intent of the Action Plan in a governance structure based on the established, hierarchical lines of authority and accountability in the bureaucracy. This connected the Minister directly to service delivery across government wherever that delivery could most effectively and efficiently take place to achieve the desired outcome. Thus, rather than setting up a mechanism for ensuring healthy policy, the Action Plan became a policy designed to achieve a single health objective through multiple complementary actions.

## The act model: what we did and how we did it

**Figure 1:**
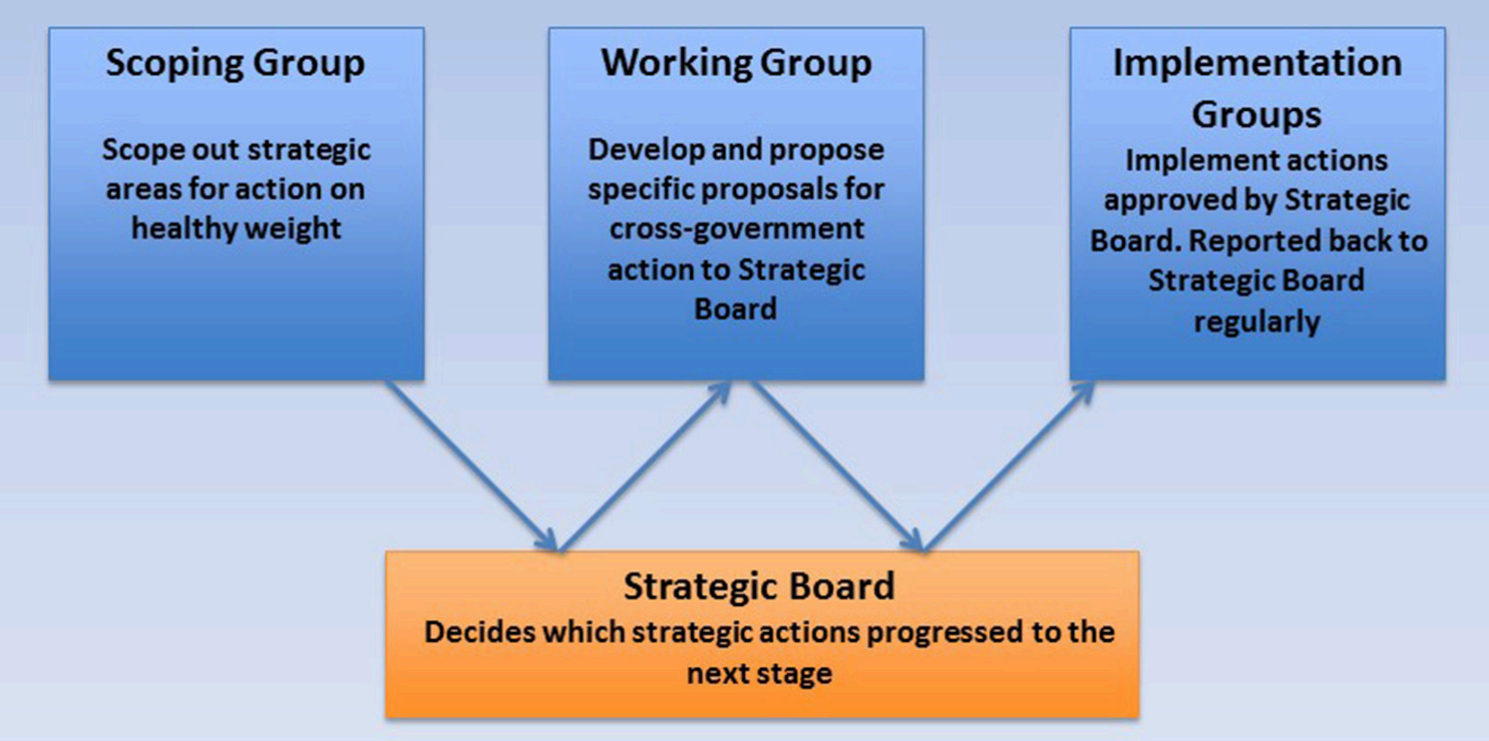
Healthy Weight Action Plan governance model.

The Action Plan developed using a phased approach in which the scope of the plan was decided, then a working group agreed actions and finally teams were convened to implement those actions. Between each of these stages the strategic board was asked to approve the progress of the work and the commencement of the next phase (Figure 1).

There were two main reasons for adopting this process. Firstly, the range of options to address obesity only became clear through the development of the Action Plan and regular executive oversight provided several points at which the feasibility and acceptability of proposals could be assessed. Without tight executive control, it would be difficult to drive progress beyond a discussion of the issue to the successful implementation of actions. Secondly, enforcing decision points between the development of proposals and their implementation provided a means of avoiding “scope creep” or prolonged discussion of proposals prior to their implementation. The implementation teams were therefore tasked to focus on how to achieve actions they had been given rather than revisiting whether these actions should be undertaken.

There is considerable public interest in obesity and politicians and public servants bring their own views regarding its causes and potential solutions to the policy process. From the outset, the prevailing view was that obesity was an issue primarily for the health agency to deal with. Consequently, causes and potential solutions remained contested throughout the process of the Action Plan development. Non-health staff tended to perceive morbid obesity, that is the upper extreme of the problem, as the primary target for action and tended to under-appreciate the difficulty of scaling intensive interventions to the population level.

Thus, a discussion was necessary across Government about the scale of the problem, the comparative role of nutrition versus exercise as proximal determinants, the importance of the environment in shaping decisions of individuals and the goals which an Action Plan might reasonably achieve. The staged approach ensured that the Executive endorsed a consensus view on the definition, extent, causes and consequences of overweight and obesity prior to discussing the question of how to address the issue. This approach was intended to ensure that the discussion of interventions was less clouded by diverging understandings of the issue to be addressed.

Having all the agency heads collectively oversight the development of the Action Plan also allowed staff time to be allocated across government using existing lines of authority and with direct accountability to the Chief Minister. It was envisaged that this high level and strongly supportive authorizing environment would streamline the process by removing the need to create a new bureaucratic structure and ensure a relatively simple process incorporating existing structures within a simple project management framework.

## Developing the action plan: making it work

Several issues were encountered during the Action Plan development process, delaying the Action Plan development by almost 18 months. One significant issue was the low level of public health literacy (15) among staff from non-health agencies. This limited the ability of agencies to identify which areas of work might best be incorporated in the Action Plan. To address this, a study was commissioned to identify projects and programs currently underway across government which impacted on levels of physical activity or nutrition. The study identified important gaps. Almost all existing programs targeted physical activity, with the exception being ACT Health-led school-based programs. Existing programs also demonstrated an emphasis on individual service delivery rather than population-wide interventions such as regulation.

Several existing strategy documents included targets relevant to reducing obesity rates, but implementation processes and reporting mechanisms associated with these documents were unclear. This suggested that there was a “baseline” level of cross-government action already taking place, but that significant value could be added by filling the gaps identified by the study. The lack of interventions that focused on improving nutrition and had population-wide reach were particularly noted. It was thought that the Action Plan might also provide a “nudge” to existing programs where high level executive support could be used to enhance reach and scale. For example, the process for including active living principles in the design of new residential developments was not well aligned with the specifications for infrastructure required by other government agencies. By targeting this relatively minor point of bureaucracy, significant improvements in urban planning could result, which in turn could contribute to addressing obesity.

The relative paucity of population-level interventions in the nutrition sphere was not entirely unexpected. Policies aimed at reducing the consumption of energy dense, nutrient poor food, and drink are likely to involve placing restrictions on the production, distribution and/or promotion of these products and therefore could be viewed as a limitation of personal liberty. Conversely, physical activity is promoted by a range of businesses such as gyms, sporting organizations, and equipment shops and thus promotion of active living could be viewed as choice-enhancing and as a business-promoting stimulus for the local economy. The survey of existing programs suggested that it had been easier for program managers to work collaboratively with the promoters of physical activity than to actively oppose the interests of food businesses. This suggested that one of the potential benefits of a whole of government plan would be to offer high level, inter-sectoral support for measures to address the food environment. Regulation, financial intervention or changes in business practice were the most difficult for individual areas to undertake, but also comprised the actions which could most influence the high proportion of the population who were overweight or obese. Therefore, the Action Plan developers concentrated in these areas.

The next step was to ensure that the working group was provided with contemporary evidence on obesity prevention initiatives which were likely to be effective in the ACT context. Thus, an independent review of the literature was commissioned (16). Whilst this provided useful guidance, determining how to apply the information gleaned from this review required extensive cross-government socialization of the findings, including individual meetings between senior Health officials and each agency head to explain the desired co-benefit methodology.

There was, in general, a degree of resistance to regulatory approaches or interventions in the commercial sector, particularly in the food environment. In most cases, non-health agencies proposed actions which were extensions of existing services, representing an expansion of effort rather than a change of approach. This reflected a level of comfort with prevailing models of Government action such as education about lifestyle choices, time-limited programs, or the provision of funding for individuals to purchase services or products. These were not interventions which had necessarily been proven to be effective or easily scalable to a population level.

Throughout the process, health officials were conscious of the need to avoid the perception of “health imperialism” that may stem from appearing to instruct other government agencies to change their business to specifically address a health issue, in this case, obesity. The preferred approach was to find actions which would have co-benefits and achieve positive health and non-health outcomes simultaneously. Improved nutrition and or physical activity levels among school children could, for example, improve educational outcomes (17, 18). It was felt that without this approach, the actions included would be resisted and not be given priority across government.

To help gain cross-government legitimacy, it was decided to allow all agencies to propose actions and then for the final plan to be based on a vote by working group members and key non-Government organizations. Nineteen actions with the strongest support and which were also supported by evidence of likely population level effect were proposed and then endorsed by the strategic board and the Chief Minister. These were then grouped into six thematic streams and included in the Action Plan (Table 1).

**Table 1:** List of actions included in the Healthy Weight Action Plan (4).

**Theme/lead agency**	**Actions**
Theme: Workplaces	1. Implement a Chief Minister's award scheme that rewards healthy workplaces and food outlets. 2. Improve the availability of healthy food and drink choices and reduce unhealthy choices at ACT Government workplaces, facilities and government-funded events.
Lead: Central agency	3. Implement a program of health risk assessments for ACT Government staff and explore options for extending this to the private sector. 4. Create new incentives for ACT workers and/or workplaces to participate in physical activity or active travel. 5. Update requirements for new commercial buildings to contain facilities which encourage physical activity and improve access to these facilities for existing buildings.
Theme: Urban planning Lead:	6. Promote and prioritize active travel through the implementation of the *Transport for Canberra* plan and master planning processes. 7. Incorporate active living principles into the Territory Plan Codes and the Territory and Municipal Services Standards for public realm design and development works.
Agency responsible for planning and environment	8. Create car parking and other incentives which encourage active travel (walk/cycle/bus) and discourage private transport for entire journeys into town centers.
Theme: Schools Lead: Education agency	9. Develop an ACT Government school food and drink policy with supporting guidelines that will mandate the implementation of the National Healthy School Canteen Guidelines in ACT Schools. 10. Improve the measurement, capacity to deliver and curriculum support for physical education in all ACT schools.
Theme: Food environment Lead:	11. Restrict the advertising of unhealthy foods within the government's regulatory control. 12.Regulate the sale of sugar-sweetened drinks. 13.Enact a mandatory code for supermarkets to require at least one checkout aisle be identified as free of energy dense, nutrient poor (EDNP) foods.
Health agency	14.Improve the availability of free drinking water in public places and food outlets.
Theme: Social inclusion Lead: Social and community services agency	15. Create new incentives for targeted populations to increase the uptake of healthy food and/or active travel options. 16.Improve awareness, skills and capability across the ACT in buying and preparing healthy food.
Theme: Evaluation Lead:	17.Develop and maintain a web-based information resource for workplaces, primary care providers and the community about opportunities to improve physical activity and nutrition levels. 18.Collect and evaluate usage and demand data about walking and cycling infrastructure to guide actions that increase use.
Health agency	19.Improve the collection and assessment of biometric data in General Practice.

This process highlighted the importance of engaging with an ongoing discussion about potential measures with the working group to balance recommendations based primarily on evidence of effectiveness on the one hand, with feasibility and political acceptability in the local context on the other.

## What we learnt

The Action Plan represents an attempt to effect whole of government action to halt the rising rates of overweight and obesity in the ACT. Lessons can be learnt from the process of developing the Action Plan, including the importance of political commitment and a clear governance framework in achieving meaningful population level action. Whilst much of what is presented here was influenced by particular temporal, political and structural factors, the lessons learnt have wider practical implications.

As there is no single cause for the rising rate of obesity, there is a need for broad action to address the issue which has in turn prompted calls for whole of Government plans (2). However, this same contested narrative about the determinants and the solutions required to address them meant that particularly strong governance structures were required to gain policy coherence to support implementation of meaningful actions (19, 20).

A specific decision was made early in the process not to formally adopt a HiAP approach, but rather to use existing formal lines of government authority, thereby increasing the chance of executives and operational areas having a common understanding of what to implement. This is possible only when there is a strong political mandate to align bureaucratic structures to focus on an issue such as obesity. This was present at the highest level of government and represented a significant “window of opportunity” to gain cross-sectoral support for the Plan (21).

Ultimately the Action Plan did conform to many of the principles of HiAP – a plan conceived by public health experts but eventually co-owned by a wider group, with an agreed way of working and a commitment to co-production for mutual benefit (4, 22–24). This highly structured approach to decision making, a limited level of health public health literacy and unfamiliarity with working in a whole of government process meant it took a significant amount of time and energy to “build the machine” prior to effective measures to address obesity being implemented. In our experience, this was at odds with the demand for action to commence rapidly once the political decision to develop the Action Plan was made, and ultimately required a degree of “bludgeoning persistence” despite a strong wish to work collaboratively. Political support, while necessary to implement a large scale plan, can sometimes be at odds with time and knowledge constraints within government. This was also observed during the formulation of the plan. Embedding a plan within the machinery of government sufficiently strongly to allow complex and sustained actions to address complex issues such as obesity may require more time than the political cycle can accommodate.

## Author contributions

Both authors were actively involved in the work described in this paper. The paper itself was conceived by PK, the first draft was written by AP and subsequently amended by PK. Both authors read and agreed to the final version as submitted and agree to be accountable for the content.

### Conflict of interest statement

The authors declare that the research was conducted in the absence of any commercial or financial relationships that could be construed as a potential conflict of interest.
